# Endoscopic ultrasonography-guided gastroenterostomy for malignant gastric outlet obstruction: Comparison between gastric and duodenal obstruction

**DOI:** 10.1055/a-2760-4544

**Published:** 2026-02-02

**Authors:** Yorick L. van de Pavert, Maronne I.D. Pheifer, Louise W.H. van Leeuwen, Rina Bijlsma, Marco Bruno, Hendrik M. van Dullemen, Paul Fockens, Akin Inderson, Willem J. Lammers, Niels G. Venneman, Rogier P. Voermans, Roy L.J. van Wanrooij, Thomas R. de Wijkerslooth, Leon M.G. Moons, Frank P. Vleggaar

**Affiliations:** 1 8124 Department of Gastroenterology and Hepatology, University Medical Center Utrecht, Utrecht, the Netherlands; 2 61363 Department of Gastroenterology and Hepatology, Martini Hospital, Groningen, the Netherlands; 3 6993 Department of Gastroenterology and Hepatology, Erasmus MC University Medical Center, Rotterdam, the Netherlands; 4 10173 Department of Gastroenterology and Hepatology, University Medical Center Groningen, Groningen, the Netherlands; 5 26066 Department of Gastroenterology and Hepatology, Amsterdam UMC, Location University of Amsterdam, Amsterdam, the Netherlands; 6 Cancer Center Amsterdam, Amsterdam UMC, Amsterdam, the Netherlands; 7 4501 Department of Gastroenterology and Hepatology, Leiden University Medical Center, Leiden, the Netherlands; 8 3231 Department of Gastroenterology and Hepatology, Medical Spectrum Twente, Enschede, the Netherlands; 9 1209 Department of Gastroenterology and Hepatology, Amsterdam UMC, Location Vrije Universiteit, Amsterdam, the Netherlands; 10 1228 Department of Gastrointestinal Oncology, Netherlands Cancer Institute - Antoni van Leeuwenhoek Hospital, Amsterdam, the Netherlands

**Keywords:** Endoscopic ultrasonography, Gastric cancer, Pancreas, Intervention EUS

## Abstract

**Background and study aims:**

It is currently unclear whether obstruction location affects clinical and procedure outcomes after endoscopic ultrasonography-guided gastroenterostomy (EUS-GE) with a lumen-apposing metal stent (LAMS) in patients with malignant gastric outlet obstruction (GOO). Therefore, we compared clinical outcomes of EUS-GE for malignant GOO located in the stomach with obstruction located in the duodenum.

**Patients and methods:**

In this nationwide, multicenter, retrospective study, we included consecutive patients who underwent EUS-GE as palliative treatment for malignant GOO. Main outcomes were clinical success, serious adverse events (SAEs) recurrence of obstructive symptoms, and LAMS dysfunction.

**Results:**

Between 2018 and 2023, 298 patients underwent EUS-GE. Clinical success was achieved in 73 of 82 patients with a gastric obstruction (94%) and in 174 of 216 patients with a duodenal obstruction (87%). No association was found between location of obstruction and clinical success (odds ratio [OR] 2.62, 95% confidence interval [CI] 0.91 to 7.52, *P* = 0.073) or SAEs (OR 0.26, 95% CI 0.06 to 1.20, *P* = 0.083). Recurrent obstructive symptoms occurred more frequently in patients with a gastric obstruction (hazard ratio 1.74, 95% CI 1.09 to 2.77, *P* = 0.020). LAMS dysfunction did not differ between the groups (7 patients [9%] with a gastric obstruction and 11 patients [5%] with a duodenal obstruction).

**Conclusions:**

In this study, EUS-GE in patients with a gastric obstruction had comparable technical and clinical efficacy and a similar safety profile to EUS-GE for duodenal obstruction. However, gastric obstruction was associated with recurrent symptoms of obstruction unrelated to LAMS dysfunction.

## Introduction

Malignant gastric outlet obstruction (GOO) is a frequently occurring consequence of advanced stages of various malignancies of the foregut, affecting up to 25% of patients with periampullary cancer and pancreatic cancer 1 2 . Symptoms of nausea, vomiting, and inability to tolerate oral intake profoundly impact quality of life 3 . Endoscopic ultrasonography-guided gastroenterostomy (EUS-GE) with a lumen-apposing metal stent (LAMS) was developed as minimally invasive treatment for malignant GOO, combining the advantages of traditional treatments: duodenal stent placement and surgical gastrojejunostomy 4 . Before the publication of the first randomised trials, several observational studies had already addressed the potential benefits of EUS-GE. Time to oral intake could be as fast as with duodenal stent placement and recurrence rates appeared to be low, comparable to the efficacy of surgical gastrojejunostomy 5 6 7 .

A recently published study found that obstruction at the level of the stomach, in contrast to the duodenum, was associated with lower odds of clinical success 8 . This finding may be explained in several ways. First, a gastric obstruction might result in recurrent obstruction due to invasion by gastric cancer 9 . Second, gastric obstruction might affect gastric motility, thereby reducing clinical efficacy of the procedure 10 . Third, patients with GOO, such as those with stomach cancer, generally exhibit a slightly more favorable prognosis than those with pancreatic cancer, thereby increasing the probability of dysfunction in the longer term 11 12 . Nevertheless, to our knowledge, the relationship between localization of the obstruction and clinical outcomes after EUS-GE has never been systematically assessed. The objective of the present study was to compare these clinical outcomes after EUS-GE for gastric versus duodenal tumor localization, in terms of clinical success, serious adverse events (SAEs), recurrence of obstructive symptoms, and LAMS dysfunction.

## Patients and methods

### Data collection

This was a Dutch nationwide, retrospective, multicenter study in which five academic and three teaching centers participated.

We included patients who were palliatively treated with EUS-GE for GOO originating from a malignant etiology. Patients were excluded who received treatment with curative intent after EUS-GE or who participated in the simultaneously recruiting randomized ENDURO-study 13 . We did not obtain informed consent from participants because all patients were deceased or terminally ill. All institutional review boards approved the study.

Patients were divided into two groups based on proximal margin of the obstruction: gastric obstruction, with the proximal margin at the level of the pylorus, antrum or corpus, and duodenal obstruction, limited to the superior, descending, horizontal, or ascending duodenum. We extracted data from the electronic health record regarding patient characteristics, including age, sex, weight, and height, American Society of Anesthesiologists (ASA) classification, World Health Organization performance status, comorbidities, and use of anticoagulants. Disease characteristics of interest included nature and severity of symptoms and etiology of obstruction. Presence of peritoneal carcinomatosis and ascites were assessed on the most recent pre-procedure computed tomography scan. Procedure data consisted of duration of EUS-GE, approach of EUS-GE, and periprocedural complications. Post-procedure data were collected on procedure outcomes, clinical success, adverse events (AEs), recurrent symptoms of obstruction, reinterventions for suspected LAMS dysfunction, confirmed LAMS dysfunction, and survival. In case of missing information, the referring hospital or general practitioner were contacted.

### Outcomes and definitions

The main outcomes of the study were clinical success, SAEs, recurrence of obstructive symptoms, and LAMS dysfunction. Clinical success was defined as a Gastric Outlet Obstruction Scoring System (GOOSS) score of 2 or higher (tolerating at least soft solid oral intake), reported by the patient after the attempted procedure 14 . Patients with technical failure and missing data on subsequent clinical success, were classified as not having achieved clinical success.

We identified perforation, LAMS maldeployment, post-procedure abdominal pain for which analgesics, presentation to the emergency department, or (prolonged) hospital stay were required, infectious complications due to the procedure, aspiration, bleeding, and cardiovascular events as procedure-related AEs. These events were retrospectively classified according to the AGREE classification 15 . SAEs were defined as AGREE 3A or higher.

Recurrence of obstructive symptoms was defined as recurrence of nausea, vomiting and/or an inability to tolerate oral intake, regardless of confirmation of LAMS dysfunction. LAMS dysfunction was defined as post-procedure obstruction due to food impaction or tissue ingrowth, or dislocation of the LAMS, confirmed with endoscopy or radiology.

Other outcomes were technical success, defined as adequate placement of the LAMS between stomach and small bowel, and AEs.

### EUS-GE procedure

All participating centers were high-volume institutions. Nevertheless, the database also included the first procedures that were performed in the participating centers.

EUS-GE was performed using the wireless technique 16 . A feeding tube was positioned downstream of the obstruction. Subsequently, a linear array echo-endoscope was positioned in the distal stomach. Saline dyed with indigo carmine or methylene blue was infused through the feeding tube to dilate the jejunum and facilitate bowel wall puncture. Antispasmodic drugs, e.g. scopolamine butyl bromide, glycopyrronium bromide, or glucagon were administered if indicated. If a sufficiently dilated bowel loop was found adjacent to the stomach, i.e. <10 mm from the stomach wall and bowel diameter approximately 40 mm, the delivery system in which the LAMS was folded was attached to the echo-endoscope. The small bowel was punctured through the stomach using electrocauterization. First, the distal flange was deployed followed with traction of the flange against the small bowel wall. Second, the proximal flange was deployed in the working channel of the echo-endoscope, and pushed out while gently retracting the echo-endoscope, resulting in deployment of the proximal flange in the stomach.

The procedure was technically successful when the dyed saline flowed back from the LAMS into the stomach and intestinal mucosa was visible through the LAMS. During hospital admission, oral intake was gradually expanded from clear liquids to solid food. Patients were generally discharged after one night if no complications occurred.

### Statistical analysis

No power analysis was done prior to this study because we attempted to include all consecutive patients who underwent EUS-GE in the participating centers.

Categorical variables are reported as absolute numbers and proportions. Continuous variables are presented as means with standard deviations or medians with 25th and 75th percentile. Differences of continuous baseline variables between the two groups were compared with the Student’s *t* -test or Kruskal-Wallis rank sum test. Categorical baseline variables were compared with a chi-square test or Fisher’s exact test.

For overall survival, patients were censored at date of death or date of last follow-up. Differences between the two groups were assessed through the log-rank test. Violation of the proportional hazards assumption was determined by assessing the Schoenfeld residuals. Median follow-up duration was calculated using the reverse Kaplan-Meier curve 17 .

The main outcomes were adjusted for potential confounders. These confounders were chosen based on differences in baseline characteristics, previously published studies, and expert opinion.

The main outcomes of clinical success and AEs were assessed with logistic regression analysis. For the outcome clinical success, age, body mass index, Eastern Cooperative Oncology Group performance status, peritoneal carcinomatosis, ascites, distant metastases, baseline GOOSS score, and LAMS diameter (15 vs 20 mm) were used as covariates 8 18 19 20 21 22 23 . With regard to AEs, age, peritoneal carcinomatosis, and ascites were included as covariates 8 18 24 . Assumptions of linearity, influential values, and multicollinearity were assessed visually.

The main outcomes of recurrence of obstructive symptoms and LAMS dysfunction were assessed as time-to-event end points. The time between EUS-GE and first day of recurrent symptoms, reintervention, or LAMS dysfunction was calculated and patients were censored at date of last follow-up. If multiple events occurred in one patient, only the first event was included in the time-to-event analyses. Because death could act as a competing event, time to recurrence of obstruction and time to LAMS dysfunction were analyzed using Fine & Gray competing risk regression analysis 25 . Both outcomes were adjusted for previous abdominal surgery, peritoneal carcinomatosis, ascites, pancreatic etiology, distant metastases, LAMS diameter, and post-procedure chemotherapy 8 18 22 24 26 27 . The outcome recurrence of obstructive symptoms was also adjusted for baseline GOOSS score. Data are visualized with cumulative incidence curves and presented as subdistribution hazard ratios (HRs) with 95% confidence intervals (CIs). For the competing risk regression analysis, the R package ‘cmprsk’ was used. Furthermore, average survival time without LAMS dysfunction at 3 and 6 months was calculated by estimating the area under the Kaplan-Meier curve until these specific time points (restricted mean survival time) 28 .

A subanalysis was done using a Fine & Gray competing risk regression model to compare patients with gastric cancer and pancreatic cancer. The same covariates as previously mentioned were included.

Missing data regarding the covariates were assumed to be missing at random and were imputed with multiple imputation by chained equations, using the “mice” package in R 29 . Predictor variables used for multiple imputation were baseline variables, covariates used in the regression analyses (excluding WHO performance status), and the outcome variables technical success, AEs, recurrence of obstructive symptoms, reinterventions for recurrence of symptoms, and LAMS dysfunction. *P* < 0.05 was considered statistically significant. All analyses were done with R software, version 4.4.0 (R Project for Statistical Computing).

## Results

**Fig. 1:**
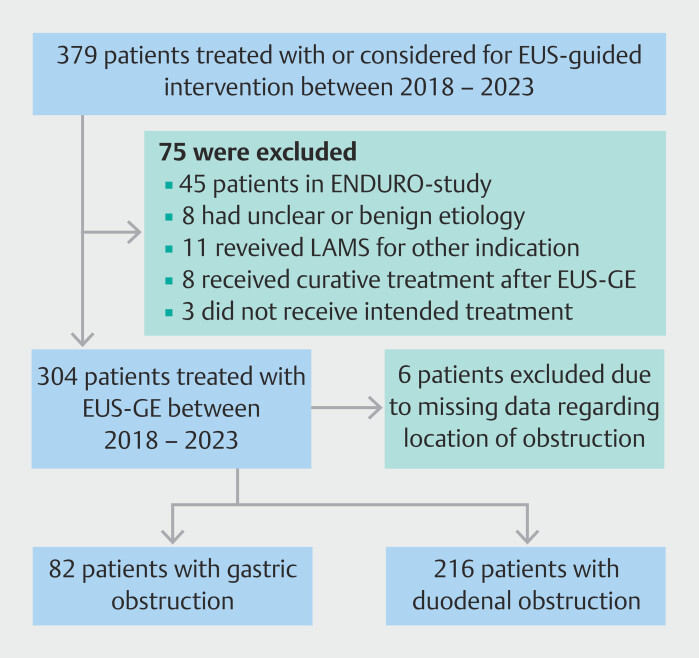
Study flowchart. EUS endoscopic ultrasonography; EUS-GE endoscopic ultrasonography-guided gastroenterostomy; LAMS lumen-apposing metal stent.

A total of 379 patients who had received or were considered for EUS-guided interventions for obstructive symptoms between January 2018 and November 2023 were screened for eligibility. Seventy-five patients did not meet our inclusion criteria ( Fig. 1 ). Of the remaining 304 patients treated for malignant GOO, six patients (2%) were excluded due to missing data regarding the location of obstruction. Eventually, 298 were included in the analysis, of whom 82 had a gastric obstruction and 216 had a duodenal obstruction. Some of these patients have been included in previously published retrospective studies 6 30 31 .

Baseline characteristics are shown in Table 1 . The most common etiology in the gastric obstruction group was stomach cancer (53 patients; 65%). Four patients with duodenal cancer were assigned to the gastric obstruction group because the proximal margin of the tumor extended above the pylorus. In the duodenal obstruction group, the most frequent cause of obstruction was pancreatic cancer (96 patients, 44%). Patients with a gastric obstruction were more frequently diagnosed with distant metastases (84% versus 63%, *P* = 0.001) and peritoneal carcinomatosis (42% versus 19%, *P* < 0.001). Ten patients (12%) with a gastric obstruction had a history of biliary drainage, compared with 107 patients (50%) in the duodenal obstruction group ( *P* < 0.001). There were no differences in American Society of Anesthesiologists classification, age, sex, or presence of ascites.

**Table:** **Table 1** Baseline characteristics of cohort.

	Gastric obstruction (n = 82)	Duodenal obstruction (n = 216)	*P* value
Age, mean (SD)	70 (11)	68 (12)	0.248
Female, n (%)	38 (46)	113 (52)	0.429
Missing	0 (0)	0 (0)	
ASA, n (%)			
1–2	27 (33)	64 (31)	0.741
3–4	54 (67)	146 (70)	
Missing	1 (1)	6 (3)	
Etiology, n (%)			
Ampullary cancer	0 (0)	8 (4)	<0.001
Biliary tract cancer	6 (7)	26 (12)	
Duodenal cancer	4 (5)	30 (14)	
Gastric cancer	53 (65)	1 (1)	
Metastatic	8 (10)	40 (19)	
Other etiology	4 (5)	15 (7)	
Pancreatic cancer	7 (9)	96 (44)	
Missing	0 (0)	0 (0)	
Presence of distant metastases, n (%)	68 (84)	136 (63)	0.001
Missing	1 (1)	1 (1)	
Presence of peritoneal carcinomatosis, n (%)	34 (42)	42 (19)	<0.001
Missing	0 (0)	0 (0)	
Presence of ascites, n (%)	22 (27)	52 (24)	0.748
Missing	0 (0.0)	1 (1)	
History of biliary drainage, n (%)	10 (12)	107 (50)	< 0.001
Missing	0 (0)	2 (1)	
GOOSS score, n (%)*			
0	33 (40)	117 (56)	0.082
1	33 (40)	68 (32)	
2	5 (6)	9 (4)	
3	11 (13)	16 (8)	
Missing	0 (0)	6 (3)	
Only patients with available data were included in the percentage calculations. *GOOSS 0 = no oral intake available, GOOSS 1 = liquids only, GOOSS 2 = soft solids, GOOSS 3 = low-residue or full diet 14 . ASA, American Society of Anesthesiologists; GOOSS, Gastric Outlet Obstruction Scoring System; SD, standard deviation.			

Patients were followed for a median of 355 days (interquartile range [IQR] 287 to 704) in the gastric obstruction group and 391 days (IQR 168 to 835) in the duodenal obstruction group. Median survival was 203 days in the gastric obstruction group (IQR 73 to 317) and 94 days (IQR 45 to 205) in the duodenal obstruction group ( *P* = 0.001).

### Technical outcomes

The (Hot) AXIOS electrocautery-enhanced stent and delivery system (Boston Scientific, Marlborough, Massachusetts, United States) was used in 77 patients (94%) in the gastric obstruction group and 198 (92%) in the duodenal obstruction group. In one patient in the duodenal obstruction group, the HANAROSTENT Plumber (M.I.Tech, Gyeonggi-Do, South Korea) was used. In five patients in the gastric obstruction group (6%) and in 17 patients in the duodenal obstruction group (8%) information about the type of stent used was missing. LAMS with diameters of 15 mm and 20 mm were used in nine patients (11%) and 62 patients (76%) in the gastric obstruction group and 33 patients (15%) and 151 patients (70%) in the duodenal obstruction group, respectively. LAMS diameter was missing in 11 patients (13%) in the gastric obstruction group and 32 patients (15%) in the duodenal obstruction group. Technical success was achieved in 77 patients (94%) in the gastric obstruction group and in 193 patients (89%) in the duodenal obstruction group ( *P* = 0.327). Additional procedure characteristics are shown in Supplementary Table 1. In the gastric obstruction group, reasons for technical failure were ascites (n = 2), gastrocolic LAMS placement (n = 1), linitis plastica (n = 1), and inability to find a proper puncture window (n = 1). The most frequently occurring reasons for technical failure in the duodenal obstruction group were LAMS maldeployment (9 patients, 4%), inability to find a proper puncture window (4 patients, 2%), and inability to pass the obstruction (4 patients, 2%). Other reasons were presence of ascites (n = 1), aspiration (n = 1), imaging processor malfunction (n = 1), hiatal hernia with intrathoracic stomach (n = 1), displacement of the target loop following puncture of the small bowel wall with the delivery system (n = 1), and identification of a second downstream obstruction (n = 1). Treatment strategies after technical failure and LAMS maldeployment are presented in Supplementary Table 2 and Supplementary Table 3.

### Clinical success

Clinical success was achieved in 73 of 82 patients with a gastric obstruction (94%, 95% CI 86–97) and in 174 of 216 patients with a duodenal obstruction (87%, 95% CI 82–91). We found no association between location of obstruction and clinical success (adjusted odds ratio [OR] 2.62, 95% CI 0.91–7.52, *P* = 0.073; Table 2 and Supplementary Table 4).

### Adverse events

SAEs occurred in two patients (2%) in the gastric obstruction group and 18 patients (8%) in the duodenal obstruction group, which was not statistically significantly different (adjusted OR 0.26, 95% CI 0.06–1.20, *P* = 0.083; Table 2 and Supplementary Table 5). In the gastric obstruction group, one patient died within 24 hours after the procedure due to arterial bleeding (1%) and one patient required a laparotomy due to gastrocolic LAMS placement (1%). In the duodenal obstruction group, six patients died following complicated LAMS placement (2%), seven patients required surgery (3%), four patients required an additional endoscopic procedure due to LAMS maldeployment (2%), and one patient required an upper endoscopy due to suspected bleeding (0.5%).

Thirty-day procedure-related mortality was 1% in the gastric obstruction group (one patient) and 2% in the duodenal group (four patients). AEs of any severity were observed in 19 patients (23%) in the gastric obstruction group and 53 patients (25%) in the duodenal obstruction group (Supplementary Table 6).

### Recurrent symptoms of obstruction and LAMS dysfunction

**Fig. 2:**
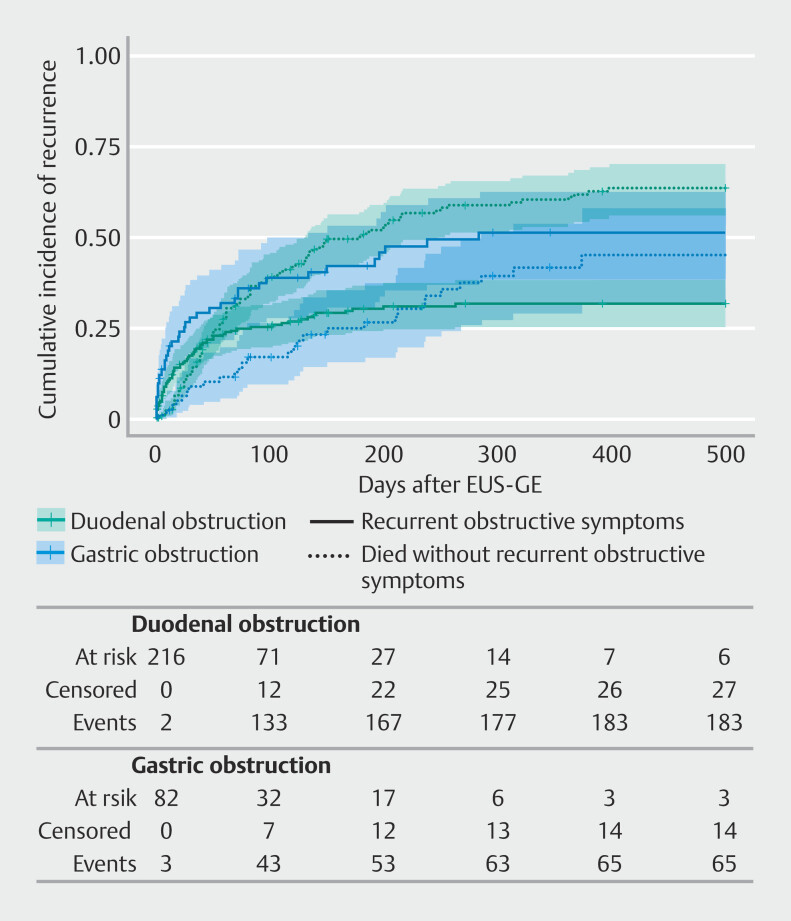
Cumulative incidence curve of recurrence of obstructive symptoms. Death is a competing event. Colored areas represent 95% confidence intervals. Tick marks indicate censored patients. EUS-GE, endoscopic ultrasonography-guided gastroenterostomy.

Thirty-eight patients (46%) with a gastric obstruction and 65 patients (30%) with a duodenal obstruction experienced recurrence of obstructive symptoms. In the multivariable competing risk analysis, gastric obstruction was associated with a higher hazard of recurrence of obstructive symptoms compared to duodenal obstruction (subdistribution HR 1.74, 95% CI 1.09–2.77, *P* = 0.020; Table 2 Fig. 2 , and Supplementary Table 7).

**Table:** **Table 2** Main outcomes.

	Gastric obstruction (n = 82)	Duodenal obstruction (n = 216)	Odds or hazard ratio	95% CI	*P* value
Clinical success, n (%)*	73 (94)	174 (87)	2.62	0.91 to 7.52	0.073
Serious adverse events, n (%) ^†^	2 (2)	18 (8)	0.26	0.06 to 1.20	0.083
Recurrence of obstructive symptoms, n (%) ^‡^	38 (46)	65 (30)	1.74	1.09 to 2.77	0.020
LAMS dysfunction, n (%) ^§^	7 (9)	11 (5)	1.36	0.48 to 3.85	0.556
*Clinical success was adjusted for age, BMI, WHO performance status, peritoneal carcinomatosis, ascites, distant metastases, and LAMS diameter (15 versus 20 mm). The estimate is the odds ratio. ^†^ Serious adverse events were adjusted for age, peritoneal carcinomatosis, and ascites. The estimate is the odds ratio. ^‡^ Recurrence of obstructive symptoms was adjusted for previous abdominal surgery, peritoneal carcinomatosis, ascites, pancreatic etiology, distant metastases, LAMS diameter, and post-procedural chemotherapy. The estimate is the subdistribution hazard ratio. **^§^** LAMS dysfunction was adjusted for the same covariates as recurrence of obstructive symptoms, with GOOSS score included as additional covariate. The estimate is the subdistribution hazard ratio. BMI, body mass index; CI, confidence interval; LAMS, lumen-apposing metal stent.					

In seven patients in the gastric obstruction group (9%) and in 11 patients in the duodenal obstruction group (5%) LAMS dysfunction was confirmed, which was not statistically significantly different (subdistribution HR 1.36, 95% CI 0.48–3.85, *P* = 0.556, Table 2 and Supplementary Table 8). Restricted mean survival time for dysfunction within the first 3 months was 86 days in the gastric obstruction group and 87 days in the duodenal obstruction group. Up to the first 6 months, restricted mean survival time was 169 days in the gastric obstruction and 171 days in the duodenal obstruction group.

In four of seven patients (57%) in the gastric obstruction group, LAMS dysfunction was caused by tissue ingrowth, two patients (29%) experienced food impaction, and one patient (14%) had insufficient luminal LAMS deployment (Supplementary Table 9).

In four of eleven patients (36%) with LAMS dysfunction in the duodenal obstruction group, this was caused by food impaction (36%), gastrojejunocolic fistula in two patients (18%), insufficient luminal deployment combined with food impaction requiring balloon dilation in one patient (9%), LAMS angulation with lumen oriented toward proximal duodenum in one patient (9%), proximal migration of LAMS in one patient (9%), tissue ingrowth in one patient (9%), and distal intestinal obstruction in one patient (9%).

Supplementary Table 10, Supplementary Table 11, Supplementary Table 12, and Supplementary Table 13 show the results of two subgroup analyses solely including patients with peritoneal carcinomatosis and distant metastases.

### Gastric cancer versus pancreatic cancer

Of the 298 included patients, 54 were diagnosed with gastric cancer (18%) and 103 with pancreatic cancer (35%). Gastric cancer, compared with pancreatic cancer, was associated with a higher hazard of recurrence of obstructive symptoms (subdistribution HR 2.31, 95% CI 1.19–4.51, *P* = 0.014, Supplementary Table 14). A secondary competing risk analysis on time to LAMS dysfunction was not performed due to the small number of events.

## Discussion

The objective of this study was to compare efficacy of EUS-GE in patients with malignant GOO caused by a gastric obstruction vs a duodenal obstruction. No differences were found in terms of clinical success or SAEs. Although recurrent obstructive symptoms were more common in the gastric obstruction group, LAMS dysfunction did not differ between groups.

Unlike the study of Pasam et al., we did not observe a lower rate of clinical success in patients with a gastric obstruction 8 . One important explanation for this contradiction is that in their study, clinical success was defined as tolerance of a low-residue diet without requiring a GOO-related reintervention before or at post-procedure day 90. This definition was stricter than the one we used: ability to tolerate at least soft solid oral intake. Consequently, we performed a univariable logistic regression analysis on the outcome of diet tolerability without reinterventions for LAMS dysfunction within 90 days *.* This also did not yield a difference between the two location groups (data not shown). Hence, based on these two distinct definitions of clinical success, it can be argued that gastric motility is not affected in such a way that an obstruction located in the stomach compromises short-term treatment effectiveness after EUS-GE.

However, as a result of their strict definition of clinical success, more than 60% of patients were excluded from analysis because of mortality or loss to follow-up within 90 days after treatment. Similarly, applying Pasam’s definition of clinical success to our sample resulted in exclusion of approximately 50% of patients from the analysis. Furthermore, in the present study, patients in the gastric obstruction group survived longer than patients in the duodenal obstruction group. This is likely due to the predominance of stomach cancer in the gastric obstruction group and pancreatic cancer in the duodenal obstruction group. To address the high attrition rate and differences in survival, we performed competing risk analyses for the outcomes recurrent symptoms and recurrent obstruction. In the analyses, death was considered a competing event to recurrence of obstructive symptoms and LAMS dysfunction, thereby taking into account the observed difference in survival between the two groups. Furthermore, we adjusted for multiple potential confounders (e.g. peritoneal carcinomatosis). The analysis yielded lower hazards for recurrence of obstructive symptoms in the duodenal group, but no difference in confirmed LAMS dysfunction was observed. This suggests that the observed difference in recurrence of obstructive symptoms between a gastric and duodenal obstruction is not necessarily explained by LAMS-related problems, but rather, by symptom recurrence due to other causes. Because the analysis was adjusted for peritoneal carcinomatosis, ascites, distant metastases, and post-procedure chemotherapy, a possible explanation for the difference in recurrence of obstruction is that a stenosis originating from a malignancy located in the stomach affects gastric motility and functioning in the longer term. For this reason, the European Society for Gastrointestinal Endoscopy guideline on therapeutic endoscopic ultrasound does not recommend EUS-GE in patients with marked infiltration of the stomach wall 10 . To assess the possible influence of infiltration in the gastric wall we did a subanalysis comparing gastric cancer with pancreatic cancer patients. Gastric cancer patients had a higher rate of recurrent obstructive symptoms, suggesting that gastric motility might play a role. However, because our study did not specifically measure gastric emptying, definitive inferences cannot be made and this hypothesis remains speculative.

What should be noted is that the high rate of recurrence of obstructive symptoms but low proportion of confirmed LAMS dysfunction likely reflects overestimation of symptoms that can be attributed to recurrence of GOO. In contrast, and although consistent with previous studies, the observed LAMS dysfunction rate is a possible underestimation of the true prevalence, because patients who experience recurrent obstructive symptoms might not undergo diagnostic work-up due to the terminal stage of their disease 7 .

In recent years, use of EUS-GE has expanded rapidly. The randomized DRA-GOO trial showed superiority of EUS-GE over duodenal stent placement in terms of reinterventions, and the GOOSE and ENDURO trials exhibited favorable results of EUS-GE compared with surgical gastrojejunostomy 32 33 34 . The present study contributes to the existing literature by demonstrating that EUS-GE is safe and effective regardless of location of obstruction. However, clinicians should be aware that patients with gastric obstruction might have an increased risk of developing recurrent obstructive symptoms, not necessarily related to LAMS dysfunction. Future studies should focus on identifying patient groups who have the highest probability of benefitting from EUS-GE.

We did not observe a difference in SAEs. However, besides one gastrocolic LAMS placement in a patient with a gastric obstruction, maldeployment solely occurred in patients with a duodenal obstruction. EUS-GE for distal duodenal obstructions might increase risk of maldeployment due to a larger distance between the stomach and jejunum, a less fixated small bowel, or more invasiveness of the primary tumor 35 .

The most important limitation of this retrospective study is the likely presence of confounding by indication. A considerable number of patients might have been missed in whom it was decided not to place the LAMS, resulting in overestimation of technical and clinical success in this cohort. Second, even though we adjusted for multiple known confounders, we could not correct for possible hidden confounders. Furthermore, the retrospective design may have weakened the association between the location of obstruction and the outcomes of interest in the analyzed models. However, we included the majority of patients who underwent EUS-GE in experienced centers in the Netherlands within the designated time period, resulting in a large homogeneous cohort from both academic and teaching hospitals. Although this study did include the first procedures that were done in the participating centers, a learning curve presumably did not affect interpretation of the results. The two groups were based on anatomical location of the obstruction and, hence, there was no specific temporal order in which the included patients were treated. The results of this study are likely to reflect real-world clinical practice and contribute to generalizability of the results to other high-volume centers. Lastly, a substantial number of patients had missing information on certain covariates or follow-up data. We attempted to minimize this by contacting the general practitioner or referring hospitals. In addition, we used multiple imputation by chained equations to account for these missing values and minimize bias.

## Conclusions

In conclusion, based on the results of this study, EUS-GE in patients with a gastric obstruction have comparable technical and clinical efficacy and a safety profile similar to EUS-GE for duodenal obstruction. However, more patients with gastric obstruction experienced recurrent symptoms of obstruction unrelated to LAMS dysfunction. This information may aid clinicians in counselling patients with malignant GOO more effectively.

## Supplementary Material

Supplementary Material Zusatzmaterial
