# Supplementary material for: Endoscopic ultrasonography-guided gastroenterostomy for malignant gastric outlet obstruction: Comparison between gastric and duodenal obstruction
Source: Endosc Int Open. 2026 Feb 2;14:a27604544. doi: 10.1055/a-2760-4544 (PMC12951034; doi:10.1055/a-2760-4544)
Supplement: Supplementary file 1 — Supplementary Material [file 10-1055-a-2760-4544_27614688.pdf]

Supplementary Table 1 Procedure characteristics of patients who underwent EUS-GE.

	Gastric obstruction (n = 82)	Duodenal obstruction (n = 216)
**Procedure duration – median minutes (IQR)**	43 (32 to 66)	45 (35 to 63)
Missing – no. (%)	21 (26)	54 (25)
**Technique – no. (%)**		
Wireless technique	74 (90)	193 (89)
Wire-guided technique	3 (4)	7 (3)
Missing	5 (6)	16 (7)
**Stent type – no. (%)**		
(Hot) AXIOS	77 (94)	198 (92)
HANAROSTENT	0 (0	1 (1)
Missing	5 (6)	17 (8)
**LAMS diameter – no. (%)**		
15 mm	9 (11)	33 (15)
20 mm	62 (76)	151 (70)
Missing	11 (13)	32 (15)

Percentages may not add up to 100 because of rounding.
EUS-GE, endoscopic ultrasonography-guided gastroenterostomy; IQR, interquartile range; LAMS, lumen-apposing metal stent.

Endosc Int Open | DOI 10.1055/a-2760-4544 | © The Author(s).

Supplementary Table 2 Treatment after technical failure.

	**Gastric obstruction (n = 5)**	**Duodenal obstruction (n = 23)**
Duodenal stent placement – no. (%)	2 (40)	6 (26)
Oral feeding tube placement – no. (%)	1 (20)	1 (4)
Laparoscopic gastrojejunostomy	-	5 (22)
Open gastrojejunostomy	2 (40)	3 (13)
Second EUS-GE attempt	-	2 (9)
Death	-	2 (9)
No treatment	-	1 (4)
Unknown	-	3 (13)

EUS-GE, endoscopic ultrasonography-guided gastroenterostomy.

Endosc Int Open | DOI 10.1055/a-2760-4544 | © The Author(s).

Supplementary Table 3 Treatment after LAMS maldeployment.

	Gastric obstruction (n = 0)	Duodenal obstruction (n = 9)
Laparoscopic gastrojejunostomy – no. (%)	-	3 (33)
Open gastrojejunostomy – no. (%)	-	1 (11)
Duodenal stent placement – no. (%)	-	2 (22)
Oral feeding tube – no. (%) placement – no. (%)	-	1 (11)
Second EUS-GE attempt – no. (%)	-	1 (11)
Death – no. (%)	-	1 (11)

EUS-GE, endoscopic ultrasonography-guided gastroenterostomy.

Endosc Int Open | DOI 10.1055/a-2760-4544 | © The Author(s).

Thieme

Supplementary Table 4 Output of logistic regression model for clinical success.

Covariate	Odds ratio	95% CI	Standard error	Degrees of freedom	*P* value
Intercept	1.19	0.03 to 46.41	1.85	127.91	0.925
Location, gastric obstruction	2.62	0.91 to 7.52	0.54	263.39	0.073
Age	1.00	0.96 to 1.03	0.02	260.07	0.929
BMI	1.11	0.98 to 1.26	0.06	131.54	0.098
WHO performance status, 3-4 vs. 1-2	1.10	0.26 to 4.74	0.72	33.76	0.895
Presence of peritoneal carcinomatosis	0.63	0.25 to 1.62	0.48	252.56	0.249
Presence of ascites	1.91	0.41 to 2.48	0.46	256.11	0.983
Presence of metastases	0.51	0.18 to 1.40	0.52	256.05	0.183
LAMS diameter, 20 mm vs. 15 mm	1.06	0.23 to 4.95	0.75	29.69	0.939
GOOSS score					
GOOSS 1	1.04	0.45 to 2.42	0.43	262.38	0.925
GOOSS 2	-	-	-	-	-
GOOSS 3	0.74	0.18 to 3.08	0.72	241.42	0.18

BMI, body mass index; CI, confidence interval; GOOSS, gastric outlet obstruction scoring system; LAMS, lumen-apposing metal stent; WHO, World Health Organization.

Endosc Int Open | DOI 10.1055/a-2760-4544 | © The Author(s).

Supplementary Table 5 Output of logistic regression model for adverse events.

Covariate	Odds ratio	95% CI	Standard error	Degrees of freedom	*P* value
Intercept	0.81	0.06 to 10.29	1.29	290.47	0.873
Location, gastric obstruction	0.26	0.06 to 1.20	0.78	290.99	0.083
Age	0.97	0.93 to 1.01	0.02	290.59	0.107
Presence of peritoneal carcinomatosis	1.79	0.61 to 5.26	0.55	290.95	0.290
Presence of ascites	0.13	0.02 to 0.98	1.05	290.91	0.047

CI, confidence interval.

Endosc Int Open | DOI 10.1055/a-2760-4544 | © The Author(s).

Supplementary Table 6 Adverse events in patients who underwent EUS-GE.

**Adverse event type**	**Gastric obstruction (n = 82)**	**Duodenal obstruction (n = 216)**
Perforation, n (%)	0 (0.0)	3 (1.4)
Maldeployment, n (%)	2 (2.4)	16 (7.4)
Abdominal pain, n (%)	6 (7.3)	12 (5.6)
Cardiovascular, n (%)	2 (2.4)	1 (1.4)
Infectious, n (%)	6 (7.3)	12 (5.6)
Aspiration, n (%)	1 (1.2)	2 (0.9)
Bleeding, n (%)	1 (1.2)	4 (1.9)
Other, n (%)*	5 (6.1)	4 (1.9)

*In the gastric obstruction group, adverse events categorized as ‘other’ were conversion of the procedure to general anesthesia due to food stasis (n =1), inability to pass the electrocautery-enhanced delivery system through the stomach wall (n =1), persistent hiccup for which haloperidol was prescribed (n =1), asymptomatic migration of a previously placed LAMS with ensuing colon perforation (n =1), and diarrhea for which medication was prescribed (n =1). In the duodenal obstruction group, other adverse events were constipation for which hospital discharge was postponed (n =1), acute tubular necrosis due to postprocedural hypotension (n =1), accidental placement of a 6- x 8-mm LAMS (n =1), and diarrhea for which medication was prescribed (n =1).
EUS-GE, endoscopic ultrasonography-guided gastroenterostomy.

Endosc Int Open | DOI 10.1055/a-2760-4544 | © The Author(s).

Supplementary Table 7 Output of Fine & Gray competing risk regression model on time to recurrence of obstructive symptoms.

**Covariate**	**Hazard ratio**	**95% CI**	**Standard error**	**Degrees of freedom**	***P* value**
Location, gastric obstruction	1.74	1.09 to 2.77	0.24	290.02	0.020
Previous abdominal surgery	1.17	0.76 to 1.80	0.22	288.63	0.462
Presence of peritoneal carcinomatosis	0.69	0.40 to 1.18	0.27	291.46	0.178
Presence of ascites	1.14	0.69 to 1.89	0.26	281.03	0.605
Etiology of pancreatic cancer	0.81	0.48 to 1.36	0.26	290.15	0.432
Presence of metastases	1.19	0.70 to 2.01	0.27	286.39	0.525
LAMS diameter, 20 mm vs. 15 mm	0.58	0.35 to 0.96	0.25	132.03	0.035
Post-procedure chemotherapy	1.09	0.66 to 1.79	0.25	156.34	0.739
GOOSS score					
GOOSS 1	1.02	0.66 to 1.58	0.22	282.26	0.924
GOOSS 2	1.00	0.40 to 2.52	0.47	282.26	0.035
GOOSS 3	1.52	0.73 to 3.16	0.37	245.70	0.727

Death was treated as a competing event.
CI, confidence interval; GOOSS, Gastric Outlet Obstruction Scoring System; LAMS, lumen-apposing metal stent.

Endosc Int Open | DOI 10.1055/a-2760-4544 | © The Author(s).

Supplementary Table 8 Output of Fine & Gray competing risk regression model on time to recurrence of obstructive symptoms.

Covariate	Hazard ratio	95% CI	Standard error	Degrees of freedom	*P* value
Location, gastric obstruction	1.36	0.48 to 3.85	0.53	284.10	0.556
Previous abdominal surgery	1.03	0.40 to 2.67	0.48	275.87	0.953
Presence of peritoneal carcinomatosis	0.21	0.04 to 1.05	0.82	288.61	0.058
Presence of ascites	2.48	0.77 to 7.99	0.59	287.67	0.126
Etiology of pancreatic cancer	0.43	0.13 to 1.40	0.60	278.75	0.160
Presence of metastases	1.11	0.38 to 3.31	0.55	286.72	0.375
LAMS diameter, 20 mm vs. 15 mm	0.32	0.13 to 0.80	0.47	239.20	0.015
Post-procedure chemotherapy	3.70	1.15 to 11.93	0.60	265.11	0.029

Death was treated as a competing event.
CI, confidence interval; LAMS, lumen-apposing metal stent.

Endosc Int Open | DOI 10.1055/a-2760-4544 | © The Author(s).

Supplementary Table 9 Specification of LAMS dysfunction.

Patient number	Location of obstruction	Time between EUS-GE and reintervention, days	Cause of LAMS dysfunction
1	Gastric obstruction	2	Insufficient LAMS deployment
2	Gastric obstruction	14	Food impaction
3	Gastric obstruction	22	Food impaction
4	Gastric obstruction	70	Tissue overgrowth
5	Gastric obstruction	202	Tissue overgrowth
6	Gastric obstruction	285	Food impaction
7	Gastric obstruction	614	Tissue overgrowth
1	Duodenal obstruction	3	Insufficient LAMS deployment
2	Duodenal obstruction	14	Food impaction
3	Duodenal obstruction	22	Colon interposition
4	Duodenal obstruction	34	Insufficient LAMS deployment
5	Duodenal obstruction	46	LAMS migration
6	Duodenal obstruction	46	Food impaction
7	Duodenal obstruction	59	Tissue overgrowth
8	Duodenal obstruction	110	Colon interposition
9	Duodenal obstruction	125	Food impaction
10	Duodenal obstruction	271	Distal intestinal obstruction
11	Duodenal obstruction	945	Food impaction

The orange line marks the distinction between LAMS dysfunction occurring before and after 90 days.
EUS-GE, endoscopic ultrasonography-guided gastroenterostomy; LAMS, lumen-apposing metal stent.

Endosc Int Open | DOI 10.1055/a-2760-4544 | © The Author(s).

Subanalysis of patients with peritoneal carcinomatosis

Supplementary Table 10 Proportion of patients with peritoneal carcinomatosis and recurrence of obstructive symptoms or LAMS dysfunction.

Outcome	Gastric obstruction (n = 34)	Duodenal obstruction (n = 42)
Recurrence of obstructive symptoms, n (%)	12 (35)	15 (35)
LAMS dysfunction, n (%)	0 (0)	2 (5)

LAMS, lumen-apposing metal stent.

Supplementary Table 11 Fine & Gray competing risks regression analysis of patients with peritoneal carcinomatosis and recurrence of obstructive symptoms or LAMS dysfunction.

Competing risk regression model	Hazard ratio	95% CI	Standard error	*P* value
Recurrence of obstructive symptoms	0.90	0.48 to 1.88	0.38	0.770

CI, confidence interval; LAMS, lumen-apposing metal stent.

Endosc Int Open | DOI 10.1055/a-2760-4544 | © The Author(s).

Subanalysis of patients with distant metastases

Supplementary Table 12 Proportion of patients with distant metastases and recurrence of obstructive symptoms or LAMS dysfunction.

Outcome	Gastric obstruction (n = 68)	Duodenal obstruction (n = 136)
Recurrence of obstructive symptoms, n (%)	33 (49)	41 (30)
LAMS dysfunction, n (%)	5 (7)	8 (6)

LAMS, lumen-apposing metal stent.

Supplementary Table 13 Fine & Gray competing risks regression analysis of patients with distant metastases and recurrence of obstructive symptoms or LAMS dysfunction.

Competing risk regression model	Hazard ratio	95% CI	Standard error	*P* value
Recurrence of obstructive symptoms	0.57	1.13 to 2.76	0.23	0.013
LAMS dysfunction	1.24	0.41 to 3.77	0.57	0.700

CI, confidence interval; LAMS, lumen-apposing metal stent.

Endosc Int Open | DOI 10.1055/a-2760-4544 | © The Author(s).

Supplementary Table 14 Output of Fine & Gray competing risk regression model on time to recurrence of obstruction, comparison of patients with gastric and pancreatic cancer.

**Covariate**	**Hazard ratio**	**95% CI**	**Standard error**	**Degrees of freedom**	***P* value**
Etiology, gastric vs. pancreatic cancer	2.31	1.19 to 4.51	0.34	151.35	0.014
Previous abdominal surgery	1.35	0.76 to 2.39	0.29	151.27	0.301
Presence of peritoneal carcinomatosis	0.52	0.24 to 1.12	0.38	136.72	0.094
Presence of ascites	1.03	0.52 to 2.03	0.34	151.38	0.933
Presence of metastases	1.04	0.51 to 2.15	0.37	152.41	0.910
LAMS diameter, 20 mm vs. 15 mm	0.49	0.24 to 0.99	0.36	127.57	0.047
Post-procedural chemotherapy	1.29	0.69 to 2.42	0.32	81.88	0.425
GOOSS score					
GOOSS 1	1.26	0.66 to 2.41	0.33	147.58	0.480
GOOSS 2	1.50	0.55 to 4.09	0.51	138.66	0.428
GOOSS 3	2.17	0.77 to 6.10	0.52	128.32	0.141

Death was treated as a competing event.
CI, confidence interval; GOOSS, gastric outlet obstruction scoring system; LAMS, lumen-apposing metal stent.

Endosc Int Open | DOI 10.1055/a-2760-4544 | © The Author(s).
